# Efficient design, accurate fabrication and effective characterization of plasmonic quasicrystalline arrays of nano-spherical particles

**DOI:** 10.1038/srep22009

**Published:** 2016-02-25

**Authors:** Farhad A. Namin, Yu A. Yuwen, Liu Liu, Anastasios H. Panaretos, Douglas H. Werner, Theresa S. Mayer

**Affiliations:** 1 Department of Electrical Engineering, Amirkabir University of Technology (Tehran Polytechnic), Tehran, Iran; 2 Intel Corporation, 2200 Mission College Blvd, Santa Clara, CA 95054, USA; 3 Department of Electrical Engineering, The Pennsylvania State University, University Park, PA 16802, USA

## Abstract

In this paper, the scattering properties of two-dimensional quasicrystalline plasmonic lattices are investigated. We combine a newly developed synthesis technique, which allows for accurate fabrication of spherical nanoparticles, with a recently published variation of generalized multiparticle Mie theory to develop the first quantitative model for plasmonic nano-spherical arrays based on quasicrystalline morphologies. In particular, we study the scattering properties of Penrose and Ammann- Beenker gold spherical nanoparticle array lattices. We demonstrate that by using quasicrystalline lattices, one can obtain multi-band or broadband plasmonic resonances which are not possible in periodic structures. Unlike previously published works, our technique provides quantitative results which show excellent agreement with experimental measurements.

A crystal is defined as a solid having an essentially discrete diffraction pattern^1^. In 1982, while studying rapidly solidified aluminum (Al) alloys, Dr. Dan Shechtman noticed that their electron diffraction pattern displayed a discrete spectrum and five-fold rotational symmetry. The discrete diffraction peaks satisfied the definition of a crystal, however, based on the *crystallographic restriction theorem*^2^, five-fold rotational symmetry could not be produced by a strictly periodic lattice. He published his results in 1984^3^ which at the time were highly controversial and met with considerable resistance in the academic community since it was a commonly held belief that all crystals possessed translational symmetry. Over the next decade, several other groups reported similar observations and this new class of crystals that lacked periodicity came to be known as quasicrystals (QCs).

In recent years there has been immense interest in the optical and electromagnetic (EM) properties of QCs^4^,^5^,^6^,^7^,^8^. Due to their unique properties, quasicrystalline geometries have been utilized in a wide range of applications such as ultra-wideband antenna arrays^9^,^10^,^11^, electronic band gap materials^12^, broadband plasmonic enhancement^8^,^13^, and surface-enhanced Raman scattering (SERS) substrates^6^. A significant challenge in the study of QCs is the lack of analytical tools to accurately and efficiently model them. Traditionally the EM properties of metamaterials and photonic crystals have been evaluated by exploiting their translational symmetry (periodicity). This approach significantly simplifies the analysis by applying periodic boundary conditions and only requiring Maxwell’s equations to be solved for one unit cell, rather than the entire structure. As noted, QCs lack translational symmetry and hence cannot be modeled accurately using periodic boundary conditions. Currently, the primary analytical method for QCs is the so-called super-cell approach. The method essentially takes a large segment of the structure and applies periodic boundary conditions to it^14^. A more efficient analysis was proposed in ref. 15 which integrates the characteristic basis function method and the adaptive integral method. Recently, a more rigorous approach to analyze the optical properties of QCs based on the cut-and-project method has been proposed^16^. The cut-and-project method constructs QC lattices as lower-dimensional slices of higher-dimensional periodic hyper-lattices^17^. Hence, Maxwell’s equations can be solved in the periodic higher-dimensional unit cell and then the solution can be projected to the physical space. This method has also been used to accurately obtain the diffraction properties of QC gratings, by essentially applying Floquet’s theorem to the higher-dimensional unit cell^18^.

A particular area of interest in recent years has been plasmonic structures based on aperiodic morphologies^5^,^6^,^8^. Their unique properties make them ideal candidates for a variety of applications such as enhanced absorption with large angular tolerance for solar cells^5^, SERS substrates^6^, and broadband plasmonic enhancement^8^. Analyzing plasmonic structures using traditional full-wave finite-difference and finite-element techniques requires considerable computational resources even for relatively simple configurations^19^. For this reason, there has been recent interest in developing more efficient analytical tools which offer solutions several orders of magnitude faster than is possible with finite-difference and finite-element methods. In the case of plasmonic nanowires, the discrete dipole approximation (DDA) method has been successfully used to model resonant light interactions with plasmonic nanowire systems^20^. In the case of spherical particles, generalized multiparticle Mie theory^21^ (GMT) can be applied to evaluate the scattering properties of arrays with arbitrary morphologies. GMT is a transfer matrix method based on an extension of Mie theory to a system with multiple spheres. This is a rigorous multiparticle approach which provides a complete solution to Maxwell’s equations and takes into account all the multipolar scattering orders. There is a vast amount of literature regarding all the analytical and numerical steps involved in implementing the GMT method^21^,^22^,^23^. GMT is far superior to the DDA method which tends to be less accurate for closely packed metal spheres in the plasmonic regime.

Furthermore, it has been shown that in order to obtain accurate results for gold (Au) spheres with diameters on the order of 100 *nm*, in the near-IR region using the DDA method, about 10^7^ dipoles per sphere are required^24^. This renders the method impractical for analysis of arrays due to the demanding computational burden.

Optical properties of aperiodic Au nanoparticle (NP) arrays where first studied in ref. 8 by using the GMT method. In ref. 8 aperiodic arrays of cylindrical Au NPs with a height of 30 *nm* and a diameter of 200 *nm* were fabricated and dark-field scattering spectroscopy was employed to characterize the scattering properties of the aperiodic arrays. For the electrodynamic calculations, the GMT method was utilized to calculate the scattering efficiencies of finite-size arrays composed of approximately 100 Au nano-spheres with radii of 100 *nm*.

Here two important points are noted regarding the results reported in ref. 8. First it was argued that since the main aim of the study was to reveal the role of array morphology, simulations based on spherical particles could be used to explain the scattering features of cylindrical NP arrays. In fact simulated and measured results were never compared directly and in some cases, there was a very little resemblance between measured and simulated patterns. The second issue had to do with the incident field used in the GMT simulations in ref. 8. While the GMT method can be applied with any physically realizable incident beam, the original paper on GMT^21^ derived complete analytical expressions for an incident plane wave and the authors later developed a very efficient open source GMT code based on an assumed plane wave excitation^25^. Using an incident plane wave considerably simplifies the computational burden in two ways. First, a plane wave has a well-known simple expansion in terms of vector spherical wave functions (VSWFs)^26^. Secondly it can be shown that for an incident plane wave, the expansion coefficients at the displaced systems differ from the primary expansion coefficients only by a constant phase term and thus does not require the application of addition theorems for VSWFs^23^. However, since a plane wave has an infinite beamwidth, it is not possible to define reflection and transmission coefficients in the usual sense when considering the analysis of finite-size arrays.

In this paper, we have overcome all the shortcomings of the previous works in this area^6^,^8^. First, utilizing a newly developed synthesis technique, we have been able to fabricate spherical NPs arrays with great accuracy whereas previous studies^6^,^8^ have relied on cylindrical NPs. Second, using the modified GMT approach introduced in ref. 13, we have been able to obtain quantitative results which can be directly compared with experimental measurements and, therefore, can be effectively used to characterize the scattering properties of QC lattices. The modified GMT approach introduced in ref. 13 was implemented for an incident wave excitation with a finite beamwidth. The incident beam was obtained by placing a circular aperture in front of a plane wave to obtain a beamwidth smaller than the array dimensions thereby avoiding diffraction by spheres at the edges of the lattice. Such a setup is very similar to realistic experimental conditions. Using the far-field expressions for scattered fields, generalized transmission and reflection coefficients were then defined based on total far-field energy fluxes.

The goal of this paper is to provide a technique that can be used to achieve quantitative analytical results for two-dimensional (2D) QC nano-spherical arrays. We were able to fabricate uniform nano-spherical arrays based on Penrose and Ammann-Beenker (A-B) 2D QCs. Furthermore utilizing the new analytical procedure developed in Ref. 13, our simulation results were directly compared to the experimental scattering measurements of fabricated samples and they showed excellent agreement. The scattering response of these nano-spherical arrays can be explained as the combination of the photonic resonances of QCs and the plasmonic resonance of the constituent Au NPs.

## Results

### Photonic Resonances of QC Lattices

Until the mid-1980s, it was presumed that all crystals possess translational symmetry. The rotational symmetry of a crystal is defined in terms of the rotational symmetry of its diffraction pattern. Thus, if the diffraction pattern of a point set is unchanged by a 2π*/n* rotation, the point set is said to possess *n*-fold rotational symmetry. Here it is important to note that according to the *crystallographic restriction theorem* it is impossible for diffraction patterns of periodic lattices in 2D or 3D space to possess rotational symmetries of order five and those greater than six^2^.

As noted, the diffraction pattern of QCs display discrete diffraction peaks with forbidden orders of rotational symmetry which means the underlying morphology of the lattice cannot be periodic. Mathematicians established the theoretical foundations for QCs in the 1960s prior to their actual discovery. As it will be shown, there is a very close relationship between QC lattices and certain aperiodic tilings of the plane. The symmetries of a crystal are the symmetries implied by its diffraction diagram. Mathematically there is a close relationship between diffraction patterns and the Fourier transform (FT).

**Figure 1:**
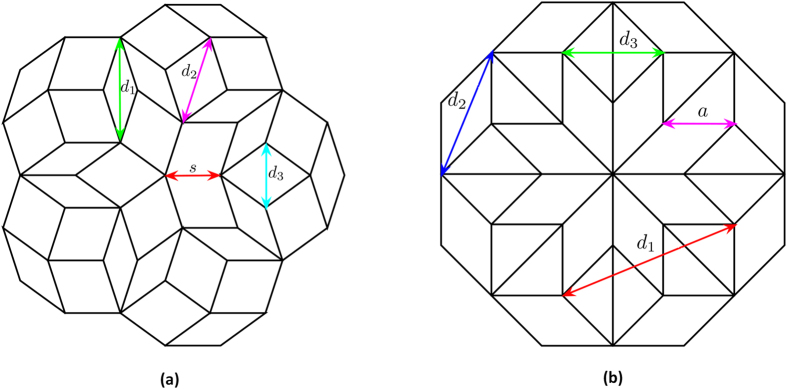
Aperiodic tiling geometries. In (**a**), a segment of the Penrose tiling with side *s* and three lattice spacings *d*_1_, *d*_2_, and *d*_3_ is displayed and (**b**) shows a segment of the A-B tiling with rhombus side *a* and three lattice spacings *d*_1_, *d*_2_, and *d*_3_ corresponding to the first three RVs.

Figure 1a shows a segment of the Penrose aperiodic tiling. The Penrose aperiodic tiling displayed in Fig. 1a is obtained by placing narrow (vertex angles *π*/5 and 4*π*/5) and wide (vertex angles 2*π*/5 and 3*π*/5) rhombi tiles of side *s* next to each other based on specific matching rules^17^. Additionally three lattice spacings are also marked in Fig. 1a, which are denoted by *d*_1_, *d*_2_, and *d*_3_. In order to obtain a point set from an aperiodic tiling, points are placed at the vertices of each tile.

Figure 1b shows a segment of an A-B aperiodic tiling. The prototile set of the A-B tiling consists of a rhombus (vertex angles *π*/4 and 3*π*/4) and an isosceles right triangle^17^ both with side *a*. As was done with the Penrose tiling, three additional lattice spacings *d*_1,_*d*_2_, and *d*_3_ have also been marked in Fig. 1b.

**Figure 2:**
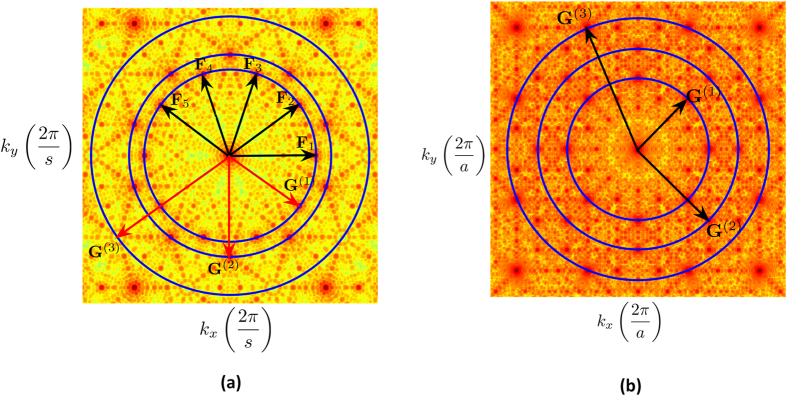
Fourier diffraction pattern (logarithmic scale) of QC lattices. In (**a**), the Fourier diffraction pattern of a Penrose lattice with tile side *s* is shown, which was obtained by taking the FT of a Penrose lattice with 776 lattice points, (**b**) shows the Fourier diffraction pattern of an A-B lattice, where the side of the rhombus-shaped prototile is *a*, which was obtained by taking the FT of an A-B lattice with 809 lattice points.

Figure 2a shows the Fourier diffraction pattern (logarithmic scale) of the Penrose QC, obtained by taking the FT of a Penrose lattice with 776 lattice points, while Fig. 2b shows the Fourier diffraction pattern (logarithmic scale) of the A-B QC, obtained by taking the FT of an A-B lattice with 809 lattice points. As it can be seen from Fig. 2a,b, the diffraction patterns of both lattices possess discrete, Bragg-like peaks. These spots can be associated with vectors in the reciprocal space, referred to as “reciprocal vectors” (RVs)^7^,^27^,^28^. It has been shown that if these RVs are indexed according to their magnitude, they can be directly associated with real space distances in the QC lattice^7^,^29^. Here it is important to note one key distinction between the reciprocal space of regular periodic crystals versus QCs. The reciprocal space of a periodic crystal is periodic, thus, it is possible to find a basis set of primitive RVs whose linear combinations form the entire reciprocal space^27^. However, in aperiodic QCs, the RVs densely fill the entire reciprocal space, and thus it is not possible to define a basis set of primitive RVs. Nevertheless, it has been proposed that a basic RV set can be constructed by omitting spots with intensity below a certain threshold^30^.

We start by considering the diffraction pattern and lattice geometry of the Penrose QC. Figure 2a shows the normalized Fourier diffraction pattern (logarithmic scale) of a Penrose lattice generated from a Penrose tiling with a tile side of *s* as shown in Fig. 1a. Additionally three lattice spacings are also marked in Fig. 1a, which are denoted by *d*_1_, *d*_2_ and *d*_3_. These lattice spacings are indexed according to their length *d*_1 _> *d*_2 _> *d*_3_ with 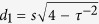 corresponding to the large diagonal of the narrow rhombus prototile while 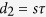 and 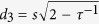 correspond to the large and small diagonals of the wide prototile respectively. An interesting property of the Penrose QC is that the different lattice spacings are related by 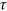, the golden ratio 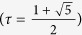. The three circles drawn in Fig. 2a have radii of 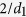, 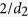, and 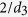 such that they coincide with the first three Bragg resonances of the Penrose QC labeled as 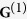, 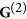, and 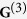. It has also been suggested that a pseudo-basis for RVs can be constructed from the most internal set of RVs^27^. This is demonstrated in Fig. 2a. Since the diffraction pattern of the Penrose QC possesses 5-fold rotational symmetry, we can define a basis set of five vectors 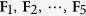 where


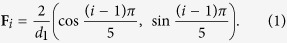


As it was noted earlier, the RVs for QCs densely fill the entire reciprocal space, thus, it is not possible to define a primitive basis set similar to what is done for periodic lattices. However, if the reciprocal space is subjected to some type of thresholding to omit spots with intensity below a certain value, then the basis set introduced in eq. 1 can be used to construct the reciprocal space^30^. For example, suppose we consider 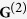 as illustrated in Fig. 2a, which can be expressed as 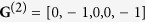 where the following vector notation has been utilized:


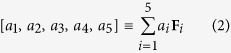


Similarly, 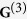 as illustrated in Fig. 2a can be expressed in the form of 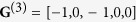.

We utilize a similar approach to analyze the diffraction pattern of the A-B QC. Figure 2b shows the normalized Fourier diffraction pattern (logarithmic scale) of an A-B lattice generated from an A-B tiling where the side of the rhombus-shaped prototile is *a*. Reciprocal vectors 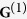, 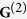, and 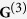 respectively correspond to lattice spacing 
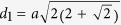
, 
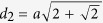
, and 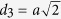 as indicated in Fig. 1b:


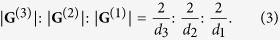


### Generalized Scattering Parameters

In ref. 8, the GMT method was used as an analytical tool to study photonic-plasmonic resonances in aperiodic Au NP arrays. GMT simulations using an incident plane wave were performed to evaluate the optical properties of finite-sized aperiodic NP arrays. Application of an incident plane wave allows calculating far-field optical quantities such as extinction, absorption, and scattering cross section of a NP array. However, these results cannot be quantitatively compared with experimental reflection and transmission measurements, and at best they can only provide a qualitative performance measure. To resolve this issue, ref. 13 reports the implementation of a new source wave excitation into the GMT framework which was obtained by placing a circular aperture of radius *a* in front of a plane wave to obtain a beamwidth smaller than the array dimensions to avoid diffraction by spheres at the edges of the lattice. Such a setup is very similar to the realistic experimental conditions shown Fig. 8a. We have outlined the key steps of this method in the Supplementary Information. A more complete description of derivations along with numerically stable implementation methods can be found in ref. 13.

The fields diffracted by the circular aperture act as the incident fields on our array (details available in Supplementary Information, eq. (S1)). The first step in application of the GMT method is the expansion of the incident field in terms of VSWFs^13^(details available in Supplementary Information, eqs (S2) to (S5)). Expansion coefficients need to be calculated in all the displaced coordinate systems defined by the sphere centers. In the case of an incident plane wave, this is a trivial matter. It can easily be shown that the expansion coefficients in the local coordinated system only differ from the primary expansion coefficients by a constant phase term^21^. However for fields scattered by a circular aperture, this is no longer true and local expansion coefficients have to be evaluated by application of vector translational addition theorems^31^,^32^ (details available in Supplementary Information, eq. (S6)).

The remainder of the process requires applying standard boundary conditions and solving for the expansion coefficients for the internal and scattered fields of all spheres. A complete description of all steps along with numerically stable algorithms to solve the resulting systems is provided in ref. 21,33. The total scattered field in the primary coordinate system can be obtained by applying vector translational addition theorems^31^,^32^ to sum all the individual scattered fields (see Supplementary Information, eq. (S9) and (S10)). However, in the far-field, much simpler asymptotic expressions for the total scattering coefficients were derived by Xu^33^ (see Supplementary Information, eq. (S11)). Using these results a generalized transmission coefficient (*T*) for finite-sized spherical arrays can be defined in terms of the total far-field energy flux relative to that of the incident field energy flux and similarly a generalized reflection coefficient (*R*) can be defined in terms of the scattered energy flux, relative to that of the incident energy flux (see Supplementary Information, eq. (S14) and (S15)).

### Experimental Setup and Measurements

**Figure 3:**
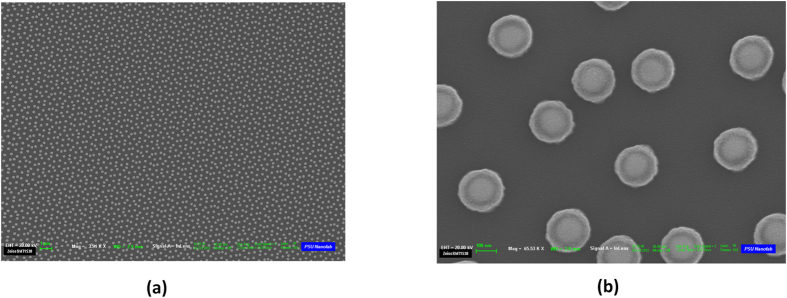
FESEM images of the Penrose array taken immediately after the conversion process. In (**a**), where the scale bar is 1 *μm*, the overall morphology of the QC geometry is displayed, whereas in (**b**), where the scale bar is 100 *μm*, the fine features of the NPs are displayed.

**Figure 4:**
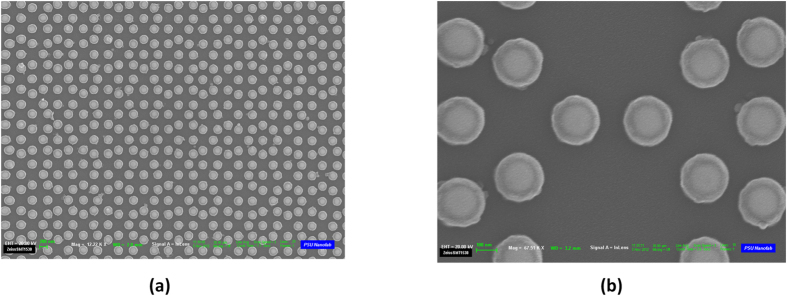
FESEM images of the A-B array taken immediately after the conversion process. In (**a**), where the scale bar is 200 *nm*, the overall morphology of the QC geometry is displayed, whereas in (**b**), where the scale bar is 100 *nm*, the fine features of the NPs are displayed.

The optimized quasicrystalline 2D spherical NP arrays were created by adopting a novel nanofabrication approach that employs electron beam lithography and subsequent thermal treatment. The details of this process are described in the Device Fabrication subsection of the Methods section. Field emission scanning electron microscope (FESEM) images were taken immediately after the conversion process and are provided in Fig. 3 for the Penrose array. Similarly, Fig. 4 shows the FESEM images taken immediately after the conversion process for the A-B array. These images confirmed that the final Au spherical NPs had the expected diameter of 135 *nm* as well as the desired aperiodic arrangements. The isotropic spherical geometry was also confirmed by the cross-sectional scanning electron microscopy (SEM). The measured minimum spacing is around 299 *nm* for both the Penrose and A-B arrays, which is in agreement with the design goals. In order to eliminate the decoherence induced by the asymmetric dielectric environment (top air and bottom substrate), both samples were immersed in an index-matching oil to create a homogeneous environment for optical measurement^34^.

The transmission of the fabricated Penrose and A-B QC arrays was measured using a UV-VIS-NIR spectrometer operating at normal incidence. This is not a straightforward process and the details are described in the Optical Characterization subsection of the Methods section. The normal incidence specular transmission spectra were background corrected by dividing the transmission intensity from an adjacent unpatterned area of equal-size.

**Figure 5:**
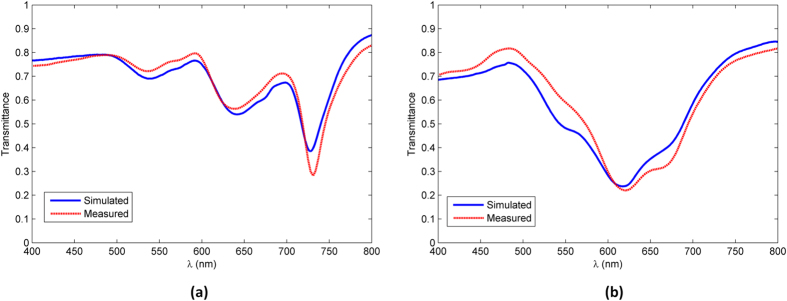
(**a**) displays the simulated (blue line) and measured (red line) transmittance spectra of the Penrose QC array, (**b**) shows simulated (blue line) and measured (red line) transmittance spectra of the A-B QC array.

Figure 5a shows the measured and simulated transmittance spectra of the Penrose QC array. The simulated transmittance values were calculated based on the method that was introduced in ref. 13 and briefly discussed in the Supplementary Information. Another point that must be considered is how to choose the appropriate distance of the array from the aperture. This topic is also discussed in the Supplementary Information.

**Figure 6:**
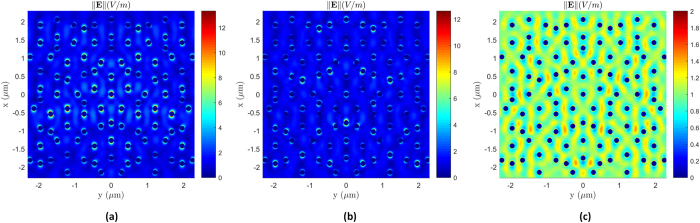
(**a**) local fields at 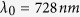 which corresponds to the first resonance, (**b**) fields at 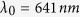, which corresponds to the second resonance, and (**c**) fields at 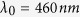 which is far removed from the resonance region.

The simulated transmittance in Fig. 5a was calculated using eq. (S14) in the Supplementary Information for a Penrose NP array consisting of 332 Au spheres, with diameters of 135 *nm* and a minimum spacing of 300 *nm*. The array was placed at a distance of 21*μm* from the aperture to ensure that there will be no diffraction caused by the peripheral elements of the array. As it can be seen from the plot, there is an excellent agreement between the predicted and measured results. To confirm the existence of localized surface plasmonic resonances we also inspect the local fields in the plane of the array. In all our simulations, we assume a linearly polarized (along *x*-axis) plane wave with a magnitude of unity. Figure 6a,b show the total local fields at *λ*_0_ = 728 *nm* and *λ*_0_ = 641 *nm*, respectively, which correspond to the first and second resonances of the Penrose lattice. As it can be seen in both cases the localized surface fields have been greatly enhanced. Figure 6c shows the fields at *λ*_0_ = 460 *nm* which is far removed from the resonance region where hardly any enhancement is observed.

**Figure 7:**
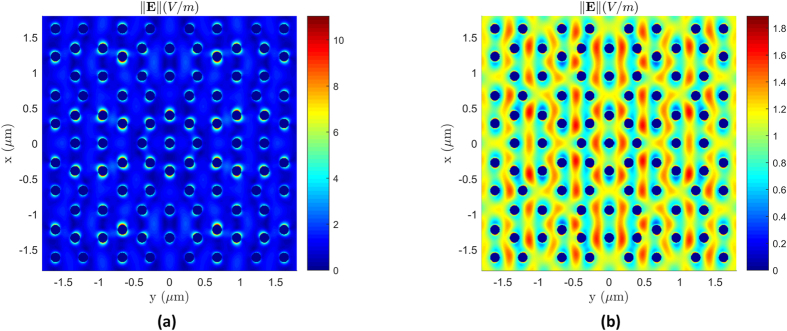
(**a**) total electric local fields in the plane of the A-B array shown at at 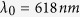 which is in the resonance region, and (**b**) fields at 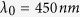 which is far removed from the resonance region.

Figure 5b shows the measured and simulated transmittance spectra corresponding to the A-B QC array. The simulated transmittance plotted in Fig. 5b was calculated using eq. (S14) in the Supplementary Information for an A-B spherical NP array consisting of 401 Au spheres, with diameters of 135 *nm* and a minimum spacing of 300 *nm*. The array was placed at a distance 22 *μm* from the aperture to ensure that there will be no edge-element diffraction^13^. As it can be seen from the plot, there is a remarkably good agreement between the predicted and measured results. As before, to confirm the existence of a localized surface plasmonic resonance, we look at the local fields in the plane of the array. Figure 7a shows the total local fields at *λ*_*0*_ = 618 *nm* which is in the resonance region of the lattice and enhanced localized surface fields can be observed. Moreover, Fig. 7b shows the fields at *λ*_*0*_ = 450 *nm* which is far removed from the resonance region and has much weaker localized fields.

**Figure 8:**
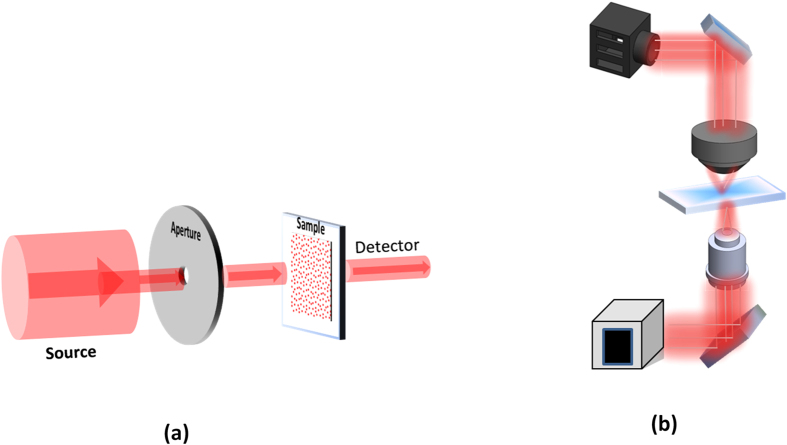
The experimental setup used for performing specular transmission measurements (**a**), and the experimental setup used for obtaining scattering maps (**b**).

**Figure 9:**
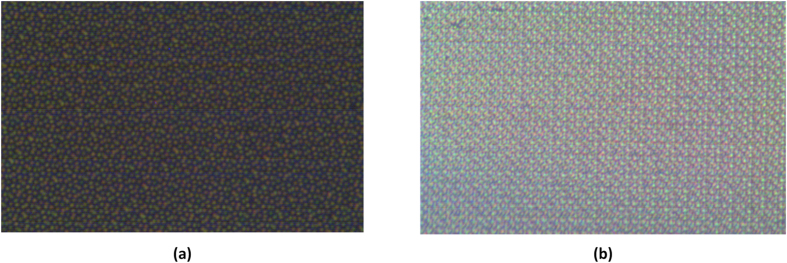
Measured scattering map of the Penrose array shown in (**a**) and for the A-B array shown in (**b**).

The plasmonic scattering response of the QC arrays was vividly confirmed by their scattering maps. The scattering maps were generated using the experimental setup shown in Fig. 8b. When illuminated by a white tungsten-halogen lamp, the QC arrays exhibit highly inhomogeneous spatial light distributions comprised of different components of light. Figure. 9a shows the scattering map of the Penrose array. As it can be seen from Fig. 9a, the scattering map is dominated by three colors and each particle is clearly visible in the map due the presence of the three distinct dip positions. Figure 9b shows the scattering map of the A-B array, where due to a single broad dip, the particles cannot be distinguished individually.

## Discussion

Diffraction properties of periodic plasmonic NP lattices have been the subject of extensive experimental and theoretical research in recent years^35^,^36^,^37^,^38^,^39^. It has been suggested that the scattering spectra of plasmonic NP lattices are composed of two types of resonances: photonic or Bragg resonances which are due to the coherent superposition of scattered fields as the incident wavelength approaches the lattice constant, and plasmonic resonances which are due to localized surface plasmon resonances of NPs^40^. Mie theory allows for the rigorous analysis of the scattering response of spheres. For an isolated sphere of radius *a* and refractive index *n*_*p*_, embedded in a nonabsorbing medium with refractive index *n*_*M*_, being illuminated by an incident plane wave with wavenumber 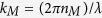, the Mie scattering coefficients *a*_*n*_ and *b*_*n*_ corresponding to *TM*^*r*^ and *TE*^*r*^ fields, respectively, are given by^41^


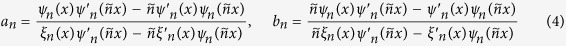


where the parameter 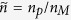 represents the normalized refractive index of the particle and the size parameter *x* is defined as *x* = *k*_*M*_*a*. The functions 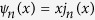 and 
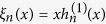
 are Riccati-Bessel and Riccati-Hankel functions, respectively. The extinction efficiency of a sphere which is defined as its extinction cross section normalized with respect to the geometric cross section 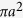 is given by^42^


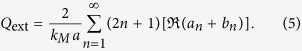


In practice, the infinite summation is truncated to a finite number of terms which is a function of the size parameter. A good approximation for the appropriate number of terms is given by the Wiscombe’s criterion^26^ as 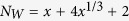.

**Figure 10:**
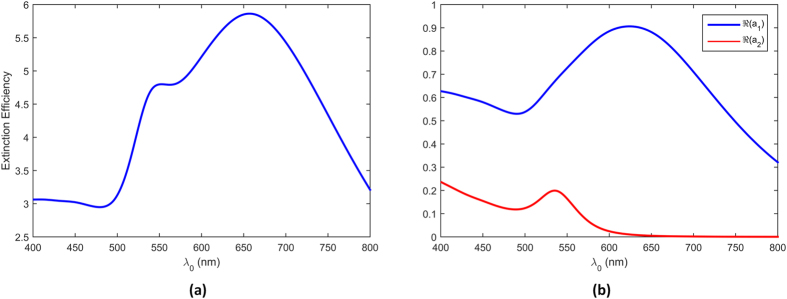
The extinction efficiency for an Au nano-sphere with a diameter of 135 *nm* is shown in (**a**) and (**b**) shows the real parts of Mie scattering coefficients *a*_1_ and *a*_2_ for the same sphere.

For plasmonic nano-spheres the scattering response is dominated by *TM*^*r*^ fields, hence for our analysis, we focus on the behavior of *a*_*n*_. For very small plasmonic spherical particles 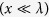, the scattering response is dominated by the dipolar mode 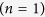. The optical extinction of such plasmonic nano-spheres has a very sharp narrow resonance with the well-known localized surface plasmon resonance condition of^41^ 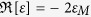. The condition is derived using the first order approximation for Bessel functions which is only valid for very small arguments. Considering the size of our spherical NPs, this approximation is no longer valid. We start by looking at the scattering response of an isolated Au nano-sphere. As noted earlier, the surrounding medium for the Au nano-spheres is 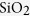 which has a relative dielectric constant of 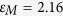 in the 400 *nm* to 800 *nm* band. Figure 10a shows the extinction efficiency of an Au nano-sphere diameter of 135 *nm* in a dielectric medium which has a relative dielectric constant of 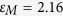. As it can be seen from the plot that there is a broad resonance with a large peak around 650 *nm* and a smaller peak around 550 *nm*. To gain a better understanding of this response, we look at the Mie scattering coefficients *a*_1_ and *a*_2_ as defined in eq 4 which correspond to dipolar and quadrupolar modes respectively. Figure 10b shows the real parts of *a*_1_ and *a*_2_ which correspond to the peaks in Fig. 10a. Thus, the broad resonance seen around 650 *nm* is due to the dipolar mode and the smaller resonance around 550 *nm* is due to the quadrupolar mode.

A rigorous theoretical analysis of the diffraction from periodic lattices has been developed by Meier *et al.*^43^. According to this theory, for a periodic lattice with lattice constant 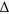, critical grating constants can be assigned to each diffraction mode. Assuming a normally incident field, the critical grating constant corresponding to the *m*-th diffraction mode is^43^


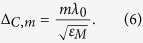


Thus at a given wavelength (*λ*_0_), for lattice constants 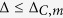, the 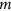-th diffraction mode is evanescent, and for 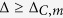 it is propagating^44^. At the boundary value, where 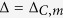, the diffraction order becomes radiating, however, it only radiates in the plane of the array (i.e. at the grazing angle). This phenomenon is of particular interest when the critical grating constant also coincides with the localized surface plasmon wavelength of the NP. When this happens, the plasmonic fields start to radiate at the grazing angle, in the plane of the array, causing stronger coupling between particles and leading to a redshift of the localized surface plasmon resonance wavelength^43^. The result is the excitation of a very sharp, so-called “hybrid photonic-plasmonic resonance^8^”. The existence of hybrid photonic-plasmonic resonances in planar periodic lattices of Au NPs was experimentally confirmed by Félidj *et al.*^45^.

At this point, it is important to make some comments regarding the nature of hybrid photonic-plasmonic resonances in periodic lattices. First we note that, as reported in ref. 45, the hybrid resonance, while very sharp has a very narrow width. This is due to the inherent relatively high-Q nature of photonic resonances that occur in periodic lattices. The second issue has to do with the number of diffraction modes that can couple to localized surface plasmons and form hybrid photonic-plasmonic resonances. From eq. (6), for a lattice with lattice constant 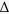, the first photonic resonance will occur at 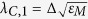, the second photonic resonance will occur at 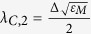, the third photonic resonance will occur at 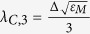 and so on. In all reported cases for periodic lattices, the hybrid photonic-plasmonic resonance mode is the result of coupling between the first grating mode and localized surface plasmon waves. Higher order modes cannot form hybrid photonic-plasmonic resonances because the resonance wavelength has changed by a factor of 2 so it falls outside the plasmonic resonance of Au nano-spheres as shown in Fig. 10a.

Hybrid photonic-plasmonic resonances can also exist in QC lattices. As noted earlier, the diffraction pattern of QCs can be effectively used to analyze their diffraction properties. Associating the RVs in the diffraction pattern with real space distances in the QC lattice is very important since it has been shown that these RVs also directly correspond to the scattering spectra of QCs^7^,^29^.

We start by considering the transmittance spectra of the Penrose QC as shown in Fig. 5a. The first three RVs of the Penrose QC, denoted by **G**^(1)^, 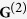, and 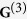 are shown in Fig. 2a which correspond to lattice spacings *d*_1_, *d*_2_, and *d*_3_ as shown in Fig. 1a. Our fabricated sample shown in Fig. 3 was based on a Penrose lattice with minimum spacing of 299*nm*. In a Penrose lattice 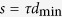, where *s* is the side length of the prototiles, thus 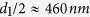, 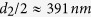, and 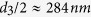. Since the surrounding medium for the Au nano-spheres is SiO_2_, which has relative dielectric constant of 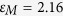, then the corresponding free space wavelengths for the first three photonic resonances are 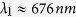, 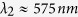, and 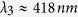. Now looking at the transmittance spectra of the Penrose QC as shown in Fig. 5a, three resonances can clearly be identified at roughly 730 *nm*, 630 *nm*, and 520 *nm*. These wavelengths are all larger than the predicted resonant wavelength, but this is to be expected since, as it was noted in ref. 7, maximum transmission occurs at the low-frequency side of each resonant band. What is more significant, however, is the ratio of these resonances in the transmission spectra which is roughly 1:0.86:0.7. This is very close to the ratio of 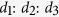. As noted earlier, in the case of periodic lattices, only the first photonic resonance mode can couple to the surface plasmon resonance. This was due to the fact that different grating orders were separated by large intervals and thus only one of them could be placed in the plasmonic resonance region of the NP. In QC lattices, however, we do not face the same obstacle. The first three photonic resonances of the Penrose QC lattice are in much closer proximity than the first three photonic resonances associated with a periodic lattice. This property in essence allows QC lattices to support multiple hybrid photonic-plasmonic resonances. This property can be clearly seen in the transmittance spectra of the Penrose QC lattice in Fig. 5a where the first two photonic resonances are highly enhanced in essence forming “hybrid photonic-plasmonic resonances” since they fall within the enhanced plasmonic region of an Au nano-sphere diameter of 135 *nm* as shown in Fig. 10a. The third photonic resonance falls outside the plasmonic resonance region which can explain its relative weakness compared to the first two, which form photonic-plasmonic resonances. The fact that these three resonances are distinguishable in the transmission spectra also sheds more light on the scattering map of the Penrose array shown in Fig. 9a which is dominated by three colors.

Next, we consider the transmittance spectra of the A-B QC as shown in Fig. 5b. Our fabricated sample depicted in Fig. 4 was based on an A-B lattice with minimum spacing of 299 *nm*. It can easily be shown that in an A-B lattice, 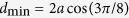, where *a* is the rhombus side as illustrated in Fig. 1b. Now, using a similar analysis as was performed for the Penrose QC, the free space wavelengths for the first three photonic resonances are 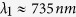, 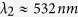, and 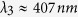. The wavelength corresponding to the third photonic resonance 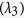 falls below the plasmonic resonance region of the Au nano-sphere as shown in Fig. 10a, and thus it cannot be detected in the transmission spectra. However, in the region between *λ*_1_ and *λ*_2_, we observe a broadband resonance where it is not easy to distinguish between the first and second photonic resonances. This fact is also demonstrated in the scattering map of the A-B array shown in Fig. 9b where due to a single broad dip, the particles cannot be distinguished individually.

In summary, it can be argued that the Penrose array provided multiple hybrid photonic-plasmonic resonances which is not feasible in periodic lattices, whereas the A-B array provided a broadband hybrid photonic-plasmonic resonance, which is larger than those reported for periodic lattices^43^,^46^.

## Methods

### Device Fabrication

The optimized quasicrystalline 2D spherical NP arrays are created by adopting a novel nanofabrication approach that employs electron beam lithography and subsequent thermal treatment. This technique combines the merits of a lithographic patterning and laser heating approach. The process begins by defining QC arrays (3 mm × 3 mm) of cylindrical amorphous-Si/Au NPs on a fused silica substrate using electron-beam lithography. Then 28 nm amorphous Si and 60 nm Au films are subsequently deposited on the patterned substrate, followed by the lift-off process. These QC cylindrical NP arrays have the desired spacing and arrangement (up to the resolution of the lithography processing). The amorphous-Si/Au cylindrical NP array can then be converted into a Au/SiO_2_ core-shell NP array by thermal oxidation at 850°*C* in 90 sccm oxygen flow for 3 hours. During this thermal treatment, Si atoms diffuse through the Au layer to form a SiO_2_ shell at the surface, and the thermodynamically unstable Au core is reshaped into a spherical geometry to reduce the surface tension^47^. This technique enables one to individually control the sphere placement, diameter, and spacing down to the nanometer scale. The final spherical Au NP diameter and inter-particle spacing can be determined entirely and precisely by the starting lithographic patterns and the evaporated Au volume. The thermal processing also offers a critical step to eliminate any possible variations in sidewall angles for cylindrical particles, and minimizes run to run variations due to uncontrollable fabrication inconsistency.

### Device Characterization

**Figure 11:**
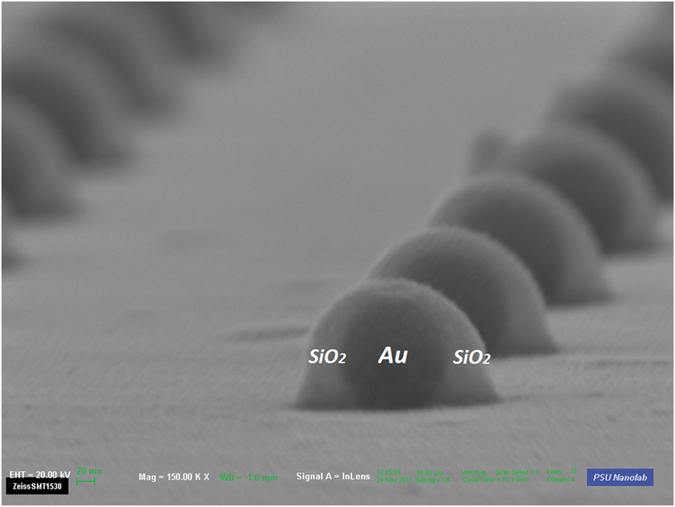
A cross sectional SEM image of fabricated core-shell nanoparticles.

In order to characterize the geometry of the fabricated structure by SEM, smaller QC arrays of 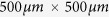 were fabricated alongside the large 3 mm × 3 mm arrays on the same substrate. A 5 nm thin layer of Iridium is sputtered on the smaller QC array before SEM to prevent the charging effect (artifacts due to electrons accumulating on the insulating substrate). FESEM images confirm that the arrays consist of Au NPs with a diameter of 135 +/- 2 nm at the desired spacing for both Penrose and AB arrays. Furthermore, the cross sectional SEM images shown in Fig. 11 demonstrate that these constituting NPs are spherical. The composition of the core shell structure has been validated on two kinds of nanowire configurations formed by the same processing using selected area TEM diffraction patterns^48^ and electron energy loss spectra (EELS)^49^. Hence, it is clear that the cores formed in these structures are single crystal Au.

### Optical Characterization

The asymmetry of the fabricated structure, with an air half space on the top and a fused silica half space on the bottom, results in decoherence of the collectively scattered waves during optical characterization^50^. This degrades both the intensity and bandwidth of the resonant features in comparison to simulation, which assumes that the particle array is embedded in a homogeneous SiO_2_ medium. To reproduce the conditions used in the simulation, the nanofabricated structure is capped with a second fused silica substrate, and index matching oil 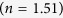 is introduced into the space between the two fused silica substrates. Normal incidence specular transmission spectra were measured using a UV-VIS spectrometer (PerkinElmer Lambda 9500 UV-VIS-NIR Spectrophotometer) with a standard detector accessory. Figure 8a illustrates the experimental setup for the transmission measurement. Unpolarized broadband white light illuminates the sample through a 2 mm circular aperture. The absolute transmission is determined by normalizing the measured transmittance of the NP array to that of the blank substrate.

## Additional Information

**How to cite this article**: Namin, F. A. *et al.* Efficient design, accurate fabrication and effective characterization of plasmonic quasicrystalline arrays of nano-spherical particles. *Sci. Rep.* **6**, 22009; doi: 10.1038/srep22009 (2016).

## Supplementary Material

Supplementary Information
