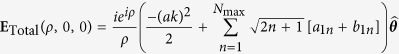# Corrigendum: Efficient design, accurate fabrication and effective characterization of plasmonic quasicrystalline arrays of nano-spherical particles

**DOI:** 10.1038/srep24655

**Published:** 2016-04-29

**Authors:** Farhad A. Namin, Yu A. Yuwen, Liu Liu, Anastasios H. Panaretos, Douglas H. Werner, Theresa S. Mayer

Scientific Reports 6: Article number: 2200910.1038/srep22009; published online: 02252016; Updated: 04292016

This Article contains a typographical error in Supplementary Equation 13:


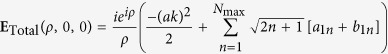


should read: