# Supplementary material for: Efficient design, accurate fabrication and effective characterization of plasmonic quasicrystalline arrays of nano-spherical particles
Source: Sci Rep. 2016 Feb 25;6:22009. doi: 10.1038/srep22009 (PMC4766558; doi:10.1038/srep22009)
Supplement: Supplementary Information [file srep22009-s1.pdf]

Efficient design, accurate fabrication and effective characterization of plasmonic quasicrystalline arrays of nano-spherical particles

Farhad A. Namin[1,+], Yu A. Yuwen[2,+], Liu Liu[3], Anastasios H. Panaretos[3,*], Douglas H. Werner[3,], and Theresa S. Mayer[3]**

[1]Department of Electrical Engineering, Amirkabir University of Technology (Tehran Polytechnic), Tehran, Iran
[2]Intel Corporation, 2200 Mission College Blvd, Santa Clara, CA 95054, USA
[3]Department of Electrical Engineering, The Pennsylvania State University, University Park, PA 16802, USA
[*]tassos@psu.edu
[**]dhw@psu.edu
[+]these authors contributed equally to this work

Supplementary Information

Generalized Scattering Parameters for Finite-Sized Spherical Arrays

Derivation of generalized scattering coefficients for finite-sized aggregates of spheres using generalized multiparticle Mie theory (GMT) was studied in Ref. 1. Here we briefly describe the approach taken and major steps involved. All mathematical functions and notations used here are in accordance with those defined in Ref. 1. The problem considered an array of L homogeneous, nonintersecting spheres, where (X^j, Y^j, Z^j) denotes the center of the jth sphere. To define reflection and transmission coefficients for the array, an incident beamwidth smaller than the sample dimensions is required to avoid diffraction. A simple way to obtain such an incident beam is to place a circular aperture in front of the array. Such a setup is very similar to realistic experimental conditions as shown in Fig. 11. Without loss of generality, we assume a z-propagating x-polarized plane wave at a fixed angular frequency ω with $e^{-i\omega t}$ time-dependence is normally incident on a circular aperture of radius a in the xy-plane $(z=0)$. It is assumed that a planar array is located at a distance d in the upper half-space $(z>0)$. The diffracted fields in the $z>0$ region will acts as the incident fields on the array and we denote them by $\mathbf{E}_{\text{inc}}$. The fields diffracted by the aperture can be derived using the Kirchhoff integral[2] as[1]

$$E_\theta = \frac{iakJ_1(ka\sin\theta)\cos\phi[j_1(\rho)+ij_2(\rho)]}{\sin\theta}$$
$$E_\phi = \frac{-iakJ_1(ka\sin\theta)\sin\phi\cos\theta[j_1(\rho)+ij_2(\rho)]}{\sin\theta} \tag{S1}$$

The first step in application of the GMT method is the expansion of the incident field in terms of vector spherical wave functions (VSWFs) $\mathbf{N}_{mn}^{(1)}$ and $\mathbf{M}_{mn}^{(1)}$ as:

$$\mathbf{E}_{\text{inc}} = -\sum_{n=1}^{\infty}\sum_{m=-n}^{n} iE_{mn}[p_{mn}\mathbf{N}_{mn}^{(1)} + q_{mn}\mathbf{M}_{mn}^{(1)}] \tag{S2}$$

In practice the expansion is truncated to $N_{\max}$ terms $(n=1,2,\ldots,N_{\max})$ which is usually determined according to the Wiscombe's criterion:[3]

$$N_W = x + 4x^{1/3} + 2 \tag{S3}$$

where $x = kR$ is the size parameter and k and R are the incident wave number and the sphere radius respectively. The expansion coefficients p_{mn} and q_{mn} are obtained by using the orthogonality of the VSWFs

$$q_{mn} = \frac{i\int_0^{2\pi}\int_0^{\pi}\mathbf{E}_{\mathrm{inc}}\cdot\mathbf{M}_{mn}^{(1)*}\sin\theta d\theta d\phi}{E_{mn}\int_0^{2\pi}\int_0^{\pi}\left|\mathbf{M}_{mn}^{(1)}\right|^2\sin\theta d\theta d\phi} \tag{S4}$$

$$p_{mn} = \frac{i\int_0^{2\pi}\int_0^{\pi}\mathbf{E}_{\mathrm{inc}}\cdot\mathbf{N}_{mn}^{(1)*}\sin\theta d\theta d\phi}{E_{mn}\int_0^{2\pi}\int_0^{\pi}\left|\mathbf{N}_{mn}^{(1)}\right|^2\sin\theta d\theta d\phi} \tag{S5}$$

Closed-form expressions for p_{mn} and q_{mn} for the incident fields shown in eq (S1) were derived in Ref. 1. Additionally, the expansion coefficients for the incident fields also have to be calculated in all the L displaced coordinate systems defined by the sphere centers. In the case of an incident plane wave, this is a trivial matter. It can easily be shown that if q^j_{mn} and p^j_{mn} denote the expansion coefficients in the jth system with its origin at (X^j, Y^j, Z^j), they only differ from primary expansion coefficients by a constant phase term.[4] However for our incident field the displaced expansion coefficients have been evaluated by application of vector translational addition theorems[5,6]as

$$\begin{aligned}
p^j_{mn} &= \sum_{\nu=1}^{\infty}\sum_{\mu=-\nu}^{\nu}\frac{E_{\mu\nu}}{E_{mn}}\left[p_{\mu\nu}A^{\mu\nu}_{mn} + q_{\mu\nu}B^{\mu\nu}_{mn}\right]\\
q^j_{mn} &= \sum_{\nu=1}^{\infty}\sum_{\mu=-\nu}^{\nu}\frac{E_{\mu\nu}}{E_{mn}}\left[p_{\mu\nu}B^{\mu\nu}_{mn} + q_{\mu\nu}A^{\mu\nu}_{mn}\right]
\end{aligned} \tag{S6}$$

where $A^{\mu\nu}_{mn}$ and $B^{\mu\nu}_{mn}$ are vector translation coefficients as defined in Ref. 1. The scattered ($\mathbf{E}^j_{\mathrm{S}}$) and internal ($\mathbf{E}^j_{\mathrm{I}}$) electric fields for the jth sphere[4] are expressed in terms of the interactive coefficients a^j_{mn}, b^j_{mn}, c^j_{mn}, and d^j_{mn}, as:

$$\mathbf{E}^j_{\mathrm{S}} = \sum_{n=1}^{N_{\max}}\sum_{m=-n}^{n} iE_{mn}[a^j_{mn}\mathbf{N}^{(3)}_{mn} + b^j_{mn}\mathbf{M}^{(3)}_{mn}] \tag{S7}$$

$$\mathbf{E}^j_{\mathrm{I}} = -\sum_{n=1}^{N_{\max}}\sum_{m=-n}^{n} iE_{mn}[d^j_{mn}\mathbf{N}^{(1)}_{mn} + c^j_{mn}\mathbf{M}^{(1)}_{mn}] \tag{S8}$$

The interactive coefficients a^j_{mn}, b^j_{mn}, c^j_{mn}, and d^j_{mn}, are obtained by applying the standard boundary conditions and solving the resulting system of equations. A detailed description of this procedure along with numerically stable methods for solving the linear system of equations can be found in Ref. 1. The total scattered electric field in the primary coordinate system with its origin at $(0,0,0)$ can be written as

$$\mathbf{E}_{\mathrm{S}}(\rho,\theta,\phi) = \sum_{n=1}^{N_{\max}}\sum_{m=-n}^{n} iE_{mn}[a_{mn}\mathbf{N}^{(3)}_{mn} + b_{mn}\mathbf{M}^{(3)}_{mn}] \tag{S9}$$

where

$$\begin{aligned}
a_{mn} &= \sum_{j=1}^{L}\sum_{\nu=1}^{\infty}\sum_{\mu=-\nu}^{\nu}[a^j_{\mu\nu}A^{\mu\nu}_{mn} + b^j_{\mu\nu}B^{\mu\nu}_{mn}]\\
b_{mn} &= \sum_{j=1}^{L}\sum_{\nu=1}^{\infty}\sum_{\mu=-\nu}^{\nu}[a^j_{\mu\nu}B^{\mu\nu}_{mn} + b^j_{\mu\nu}A^{\mu\nu}_{mn}]
\end{aligned} \tag{S10}$$

To define generalized scattering coefficients, we consider the far-field energy fluxes. Asymptotic expressions for the total scattering coefficients were derived by Xu[7] as

$$\begin{aligned}
a_{mn} &= \sum_{j=1}^{L}\exp(-ik\Delta^j)a^j_{mn}\\
b_{mn} &= \sum_{j=1}^{L}\exp(-ik\Delta^j)b^j_{mn}
\end{aligned} \tag{S11}$$

where $\Delta^j = X^j \sin\theta\cos\phi + Y^j \sin\theta\sin\phi + Z^j \cos\theta$ and (X^j, Y^j, Z^j) denotes the center of the jth sphere. It can be shown that for $\theta = 0$, the scattered far-field has the form

$$\mathbf{E}_{\mathrm{S}}(\rho,0,0) = \frac{ie^{i\rho}}{\rho}\sum_{n=1}^{N_{\max}}\sqrt{2n+1}[a_{1n}+b_{1n}]\hat{\boldsymbol{\theta}} \tag{S12}$$

The total far-field for $\theta = 0$ can be expressed as the sum of incident and scattered fields according to

$$\mathbf{E}_{\mathrm{Total}}(\rho,0,0) = \frac{ie^{i\rho}}{\rho}\Big(\frac{-(ak)^2}{2} + \sum_{n=1}^{N_{\max}}\sqrt{2n+1}[a_{1n}+b_{1n}]\Big) \tag{S13}$$

A generalized transmission coefficient (T) can now be defined in terms of the total far-field energy flux relative to that of the incident field energy flux for $\theta = 0$

$$T = \left|\frac{\mathbf{E}_{\mathrm{Total}}(\rho,0,0)}{\mathbf{E}_{\mathrm{inc}}(\rho,0,0)}\right|^2 = \left|1 - \frac{2}{(ak)^2}\sum_{n=1}^{N_{\max}}\sqrt{2n+1}[a_{1n}+b_{1n}]\right|^2 \tag{S14}$$

Similarly a generalized reflection coefficient (R) can be defined in terms of the scattered energy flux for $\theta = \pi$ relative to that of the incident energy flux for $\theta = 0$

$$R = \left|\frac{\mathbf{E}_{\mathrm{S}}(\rho,\pi,0)}{\mathbf{E}_{\mathrm{inc}}(\rho,0,0)}\right|^2 = \left|\frac{2}{(ak)^2}\sum_{n=1}^{N_{\max}}(-1)^n\sqrt{2n+1}[a_{1n}-b_{1n}]\right|^2 \tag{S15}$$

Appropriate Distance of the Aperture from the Array

Another important point to consider in this problem is choosing the appropriate distance of the aperture from the array. As noted the purpose of using an aperture was obtaining an incident field with a finite beamwidth smaller than the array dimensions to avoid diffraction by spheres at the edges of the lattice. However if the array is placed too far from the aperture the beamwidth might exceed the array dimensions. Hence it is important to find the appropriate distance of the aperture from the array (d) such that diffraction of the fields at the periphery of the array is avoided.

Considering the expression of the incident fields in Eq. (S1), a simple way to determine the appropriate distance is using the first zero of the $\frac{J_1(ka\sin\theta)}{\sin\theta}$ term which approximately occurs at $ka\sin\theta_0 \approx 3.832$. Denoting the set of spheres on the periphery of the array by S and their spherical coordinates by (r^s, θ^s, ϕ^s) where $s \in S$, we define $\alpha \equiv \underset{s\in S}{\operatorname{argmin}}\, \theta^s$. The condition

$$\theta^\alpha \leq \theta_0 \quad , \quad d = r^\alpha \cos(\theta^\alpha) \tag{S16}$$

ensures that the incident fields at the peripheral elements of the array are zero or much diminished compared to the incident fields on the interior elements of the array.

References

1. Namin, F., Wang, X. & Werner, D. H. Reflection and transmission coefficients for finite-sized aperiodic aggregates of spheres. *J. Opt. Soc. Am. B* **30**, 1008–1016 (2013).

2. Jackson, J. D. *Classical Electrodynamics* (Wiley, New York, NY, 1975).

3. Bohren, C. F. & Huffman, D. R. *Absorption and Scattering of Light by Small Particles* (Wiley-VCH, Weinheim, Germany, 2004).

4. Xu, Y. L. Electromagnetic scattering by an aggregate of spheres. *Applied Optics* **34**, 4573–4588 (1995).

5. Stein, S. Addition theorems for spherical wave functions. *Q. Appl. Math.* **19**, 15–24 (1961).

6. Cruzan, O. R. Translational addition theorems for spherical vector wave functions. *Q. Appl. Math.* **20**, 33–40 (1962).

7. Xu, Y. L. Electromagnetic scattering by an aggregate of spheres: Far field. *Appl. Opt.* **36**, 9496–9508 (1997).
